# Validation of a multi-parameter algorithm for personalized contrast injection protocol in liver CT

**DOI:** 10.1186/s41747-024-00492-8

**Published:** 2024-10-09

**Authors:** Hugues G. Brat, Benoit Dufour, Natalie Heracleous, Pauline Sastre, Cyril Thouly, Benoit Rizk, Federica Zanca

**Affiliations:** 1 Institut de Radiologie de Sion, Groupe 3R, Sion, Switzerland; 2 GE Healthcare, Buc, France; 3 Centre d’Imagerie de Fribourg, Groupe 3R, Fribourg, Switzerland

**Keywords:** Abdomen, Body composition, Contrast media, Liver, Multidetector computed tomography

## Abstract

**Background:**

In liver computed tomography (CT), tailoring the contrast injection to the patient’s specific characteristics is relevant for optimal imaging and patient safety. We evaluated a novel algorithm engineered for personalized contrast injection to achieve reproducible liver enhancement centered on 50 HU.

**Methods:**

From September 2020 to August 31, 2022, CT data from consecutive adult patients were prospectively collected at our multicenter premises. Inclusion criteria consisted of an abdominal CT referral for cancer staging or follow-up. For all examinations, a web interface incorporating data from the radiology information system (patient details and examination information) and radiographer-inputted data (patient fat-free mass, imaging center, kVp, contrast agent details, and imaging phase) were used. Calculated contrast volume and injection rate were manually entered into the CT console controlling the injector. Iopamidol 370 mgI/mL or Iohexol 350 mgI/mL were used, and kVp varied (80, 100, or 120) based on patient habitus.

**Results:**

We enrolled 384 patients (mean age 61.2 years, range 21.1–94.5). The amount of administered iodine dose (gI) was not significantly different across contrast agents (*p* = 0.700), while a significant increase in iodine dose was observed with increasing kVp (*p* < 0.001) and in males *versus* females (*p* < 0.001), as expected. Despite the differences in administered iodine load, image quality was reproducible across patients with 72.1% of the examinations falling within the desirable range of 40–60 HU.

**Conclusion:**

This study validated a novel algorithm for personalized contrast injection in adult abdominal CT, achieving consistent liver enhancement centered at 50 HU.

**Relevance statement:**

In healthcare’s ongoing shift towards personalized medicine, the algorithm offers excellent potential to improve diagnostic accuracy and patient management, particularly for the detection and follow-up of liver malignancies.

**Key Points:**

- The algorithm achieves reproducible liver enhancement, promising improved diagnostic accuracy and patient management in diverse clinical settings.
- The real-world study demonstrates this algorithm’s adaptability to different variables ensuring high-quality liver imaging.
- A personalized algorithm optimizes liver CT, improving the visibility, conspicuity, and follow-up of liver lesions.

**Graphical Abstract:**

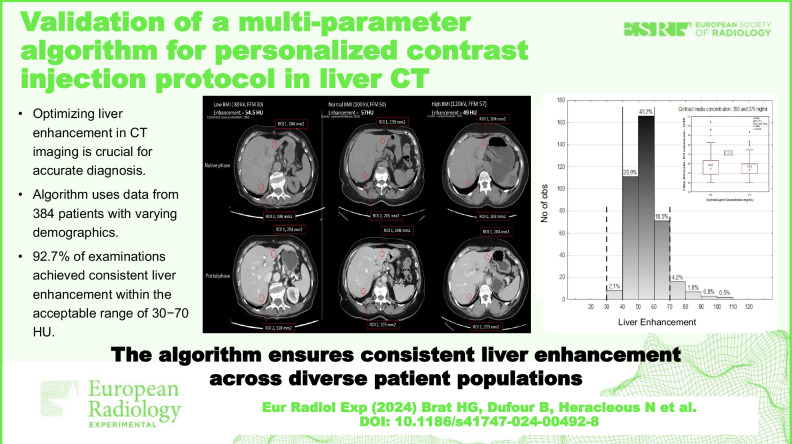

## Background

Detection and characterization of hypervascular and hypovascular hepatic lesions are paramount for the accurate management of liver malignancy. The intravenous administration of contrast agents play a pivotal role in enhancing the conspicuity of liver lesions in multidetector computed tomography (CT) imaging. Individualized contrast protocols play are crucial in optimizing diagnostic imaging outcomes in CT scans. Tailoring the contrast administration to each patient’s specific characteristics, such as body weight, renal function, and underlying health conditions, ensures both patient safety and imaging efficacy. By carefully adjusting the contrast dosage and administration parameters, clinicians can achieve optimal contrast enhancement, enabling clearer visualization of anatomical structures and pathological abnormalities. This individualized approach not only minimizes the risk of adverse reactions but also enhances diagnostic accuracy, ultimately leading to improved patient care and treatment decisions.

Personalized contrast injection protocols have emerged as a valuable approach to optimize lesion visualization and improve diagnostic accuracy [1–12]. However, it is uncertain whether these personalized protocols are generalizable, as they are often developed based on local settings, using a specific scanner type, kVp, injection rate, and contrast agent concentration, and do not account for a target liver enhancement as a reference image quality across all patients, which has been reported to be 40–60 HU [13, 14].

At our institution, we earlier developed a generalizable algorithm for personalized contrast injection for liver CT, which considers different scanner types, contrast agent concentrations, and kVp settings and uses fat-free mass (FFM) as body composition parameters to predict contrast dose [15]. In this earlier study, we indeed compared total body weight and FFM as predictors of contrast enhancement. Our findings identified FFM as the superior predictor in this regard. FFM was then chosen as the body parameter in our algorithm, established through simulations. The algorithm was engineered to integrate any desired image quality, expressed as a liver enhancement value. Its adaptability extends across diverse patient habitus, scanner types, imaging parameters, and contrast agent concentrations. While the algorithm’s validity has been established through simulations, clinical validation in real-world settings remains crucial.

This study aimed to validate the generalizability of this algorithm in clinical settings for an automated administration of personalized intravenous contrast dose in adult CT examinations and enable a reproducible liver enhancement centered at 50 HU.

## Methods

The study was approved by the Commission Cantonale d’Ethique de la Recherche (CCER) ((Req-2020-00770).

### Patient data collection

From September 2020 to August 2022, CT data from consecutive adult patients were prospectively collected at our multicenter premises, as later described. Inclusion criteria consisted of an abdominal CT referral for cancer staging or follow-up, as well as liver lesion characterization or follow-up examinations. Patients with fatty liver (< 40 HU [16]), cirrhosis (surface and parenchymal regenerative, siderotic or dysplastic nodularity, signs of portal hypertension [17]) or fibrotic liver changes (wedge-shaped regions of hypoattenuation on unenhanced CT, hypoattenuating on the arterial and portal venous phases [18]) and hemochromatosis (marked homogeneous increase in native liver density (> 75 [19], with portal vessels and hepatic veins of low attenuation relative to the liver on unenhanced CT [20, 21]) were excluded, due to the dysmetabolic impact on parenchymal attenuation. All patients underwent a hepatic dynamic CT, including at least one unenhanced and a contrast-enhanced portal venous phase scanning.

### Scanning protocol and contrast injection

Before this study, the adult (> 16 years of age) CT acquisition protocols of nine CT scanners were harmonized and optimized, based on clinical indication and body mass index (BMI) [22, 23]. The scanners, installed at 8 centers of the Swiss Groupe 3R (Réseau Radiologique Romand, https://www.groupe3r.ch/), were: 7 Revolution EVO, one installed in 2017, one in 2018, 1 one 2019 and 4 in 2020, equipped with True Fidelity^TM^ deep-learning-based reconstruction and 2 Revolution Frontier, 1 installed in 2018 and 1 in 2020, equipped with ASIR-V^TM^ iterative reconstruction and upgraded to True Fidelity^TM^ since April 2022, all of them manufactured by GE Healthcare (Chicago, Illinois, United States).

Automatic tube current modulation of the x-ray tube was used for all scanners. Concerning the tube potential, if True Fidelity was present on the scanner, several protocols were set up based on patient BMI and using different kVp values (80, 100, or 120 kVp), with 100 kVp being the standard for normal-size patients. On the Revolution Frontier scanners equipped with the ASIR-V reconstruction, a fixed 100 kVp was generally used. The other scanning parameters were detector configuration 64 × 0.625 mm, pitch 1, collimation 40 mm, gantry rotation time 0.4–0.5 s, and large body field of view. The True Fidelity setting was “High” while the kernel was selected as “Standard”. The ASIR-V strength was also 50% with a standard kernel.

Iopamidol (Iopamiro^TM^, Bracco, Milan, Italy) 370 mgI/mL or Iohexol (Accupaque^TM^, GE Healthcare) 350 mgI/mL contrast agent and saline flush (30 mL) were injected with the same dual mechanical power injector (Medrad Stellant class IV, Bayer Healthcare, Leverkusen, Germany) at a variable injection rate (arterial phase at 20 s, portal phase at 40 s, to reach the plateau phase at 80 s). An antecubital 21 G 32-mm plastic intravenous catheter was used.

All examinations were performed using a real-time low-dose (100 kVp, 40 mA, 0.5 s) bolus-tracking program (Smart Prep; GE Healthcare) initiated 15 s after contrast injection to determine the CT acquisition starting time. Twenty-five seconds after reaching a threshold of 120 HU in a region of interest placed in the supradiaphragmatic aorta, the equilibrium phase was considered to be initiated, and the portal phase acquisition was performed at 80 s after the injection started.

All CT scanners were connected to a dose monitoring system (latest version, DoseWatch 3.2.4, GE Healthcare), equipped with a contrast management module for contrast type, injection rate, volume, and concentration data collection.

### Algorithm for personalized contrast injection

A web interface implementing our algorithm was developed to assess—before the examination—the personalized contrast dosing for optimal and reproducible liver enhancement. The interface includes data imported from the radiology information system (patient name and date of birth, examination date, and accession number) and data to be filled in by the radiographer before the examination; patient FFM [15], imaging center (to be able to identify the scanner type), kVp used for the examination, contrast agent concentration and imaging phase of interest (portal or arterial). The FFM was measured before each examination using a BIA-ACCTM impedance meter (BioTekna, Marcon, Venice, Italy). Two skin electrodes at a measured distance of 5 cm were placed on the patient’s third metacarpal and third metatarsal level. After inserting these data, the algorithm gives as output the contrast volume and the injection rate to be used for the patient examination. The calculated contrast volume and injection rate could be directly inserted into the CT console, which controls the injector. The algorithm was set up to obtain the desired optimal diagnostic level of 50 HU for the portal phase liver enhancement [7]. Values < 30 HU were considered insufficient for accurate diagnosis due to reduced visibility and conspicuity of low-contrast liver lesions [10].

Note also that the algorithm was set up to account for an upper and lower contrast dose limit following each contrast agent’s intended use: for Iopamidol, the minimum, and maximum contrast dose range was 0.5 to 2 mL/kg of iodine; for Iohexol, the minimum and maximum contrast dose range was 30–60 g of iodine. Preliminary data analysis showed that the lower limit of 30 g of iodine for Iohexol barred the algorithm’s potential to suggest low-contrast doses, with most of the livers being overenhanced. In accordance with the contrast agent producer, we adapted the Iohexol lower/upper limits to be the same as for Iopamidol, receiving approval by the Ethical Committee.

### Quantitative image quality evaluation: contrast-enhancement index of normal parenchyma

To validate the implemented algorithm and assess whether the desired image quality of the liver was centered around the target of 50 HU (acceptable range 30–70; HU, desirable range 40–60 HU), liver parenchymal enhancement was measured using a clinical workstation (Advantage Windows Server version 3.0 and 3.2, GE Healthcare) by one senior radiologist and a trained medical physicist. Circular regions of interest (ROIs) of similar size (range 20–30 mm in diameter), locations (liver segments III and VI), and scan table position were drawn on unenhanced and portal phase 3-mm thickness axial reconstructions, as described in a previous publication [15].

The level of enhancement was determined by the contrast-enhancement index (CEI) of normal parenchyma (CEI_NP_) [15], consisting of attenuation difference (HU) between portal and unenhanced parenchyma.

$${{{{{\rm{CEI}}}}}}_{{{{{\rm{NP}}}}}}=\sum {ROI}\frac{{HUportal}}{N}-\sum {ROI}\frac{{HUunenhanced}}{N}$$

with *N* being the number of ROIs.

### Statistical analysis

Patient characteristics were analyzed per BMI, FFM, age, and sex.

The total iodine dose (in grams) administered to a patient was determined by calculating the product of the injected volume (measured in mL) and the contrast agent concentration (measured in gI/mL), as obtained from the DoseWatch software. Boxplots for the administered iodine dose were generated. CEI_NP_ descriptive statistics (median, minimum, maximum, 25% lower quartile, and 75% upper quartile were performed, and histograms were generated. All data were stratified also per contrast type, kVp, and sex. The Wilcoxon rank-sum test and the Kruskal–Wallis were used to assess significant differences with *p*-values lower than 0.05 considered to be significant, as appropriate. Further post-hoc tests (pairwise Wilcoxon rank-sum tests with a correction for multiple comparisons) were performed to identify differences between groups.

## Results

### Patient population

The study population consisted of 384 patients. Data distribution is reported in Table 1.

**Table 1:** Data on patients, contrast dose, and image quality

Timeframe	Number of cases	Male (%)/Females (%)	Age, years (range)	BMI, range	FFM, kg (range)	Contrast dose, mgI (range)	Contrast volume injected, mL (range)	Image quality, HU (range)
September 2020 to August 2022	384	43.5/56.5	61.2 (21.1–94.5)	26.1 (16.5–50.2)	52.6 (23.0–128.0)	26.9 (13.3–46.0)	73.8 (36.1–124.3)	55.8 (40.0–105.0)

Data are mean values (min–max)

*BMI* Body mass index, *FFM* Fat-free mass, *TBW* Total body weight

### Total amount of Iodine dose

**Fig. 1:**
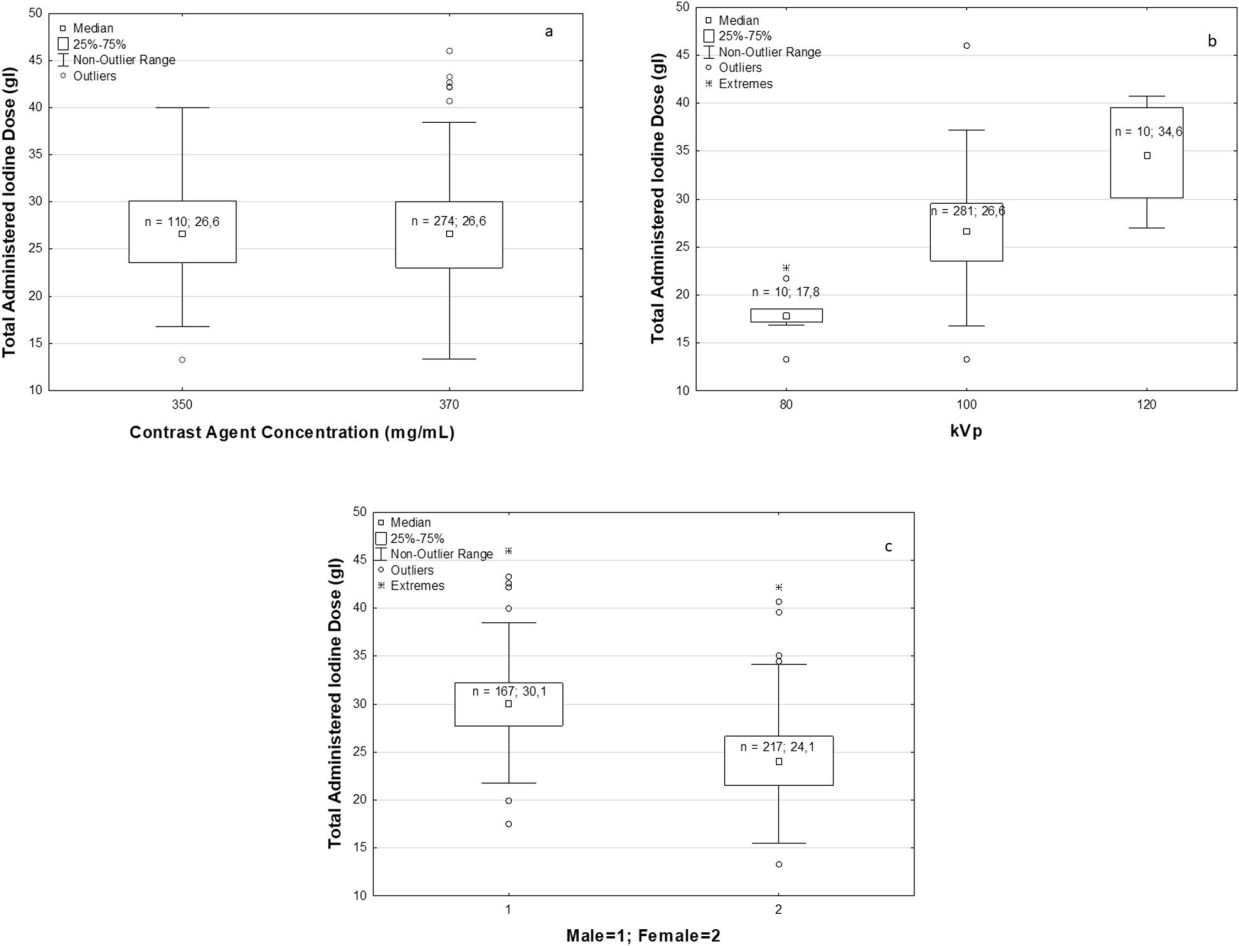
Boxplots of the total administered iodine dose (gI) stratified by contrast concentration (**a**), kVp (**b**), and sex (**c**). *N* is the number of cases, and the median value per boxplot is shown

**Fig. 2:**
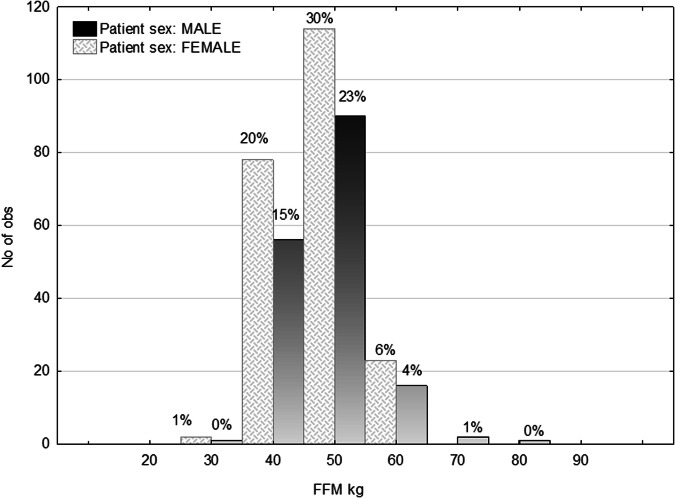
Histogram distribution of measured free-fat mass (FFM) in kg for male and female populations. No. of obs, number of observations

The median iodine dose administered over the population was 26.6 gI, with 25% lower quartile and 75% upper quartile being 23.2 and 30.1 gI, respectively. Figure 1a reports the total amount of administered iodine dose (gI) stratified per contrast concentration; no significant difference was observed (*p* = 0.700). The central graph (Fig. 1b) shows the same data stratified per tube potential. As expected from the algorithm, the amount of iodine dose increases with the kVp (*p* < 0.001). The post-hoc tests showed a *p*-value < 0.001 for each pair of groups. The lower graph (Fig. 1c) shows the stratification per sex, and it is observed that the female population received a significantly lower (*p* < 0.001) amount of iodine dose than the male one. This is explained by looking at Fig. 2, where it is clear that females typically have a lower FFM than males (*p* < 0.001).

#### Quantitative image quality evaluation: contrast-enhancement index of normal parenchyma

**Fig. 3:**
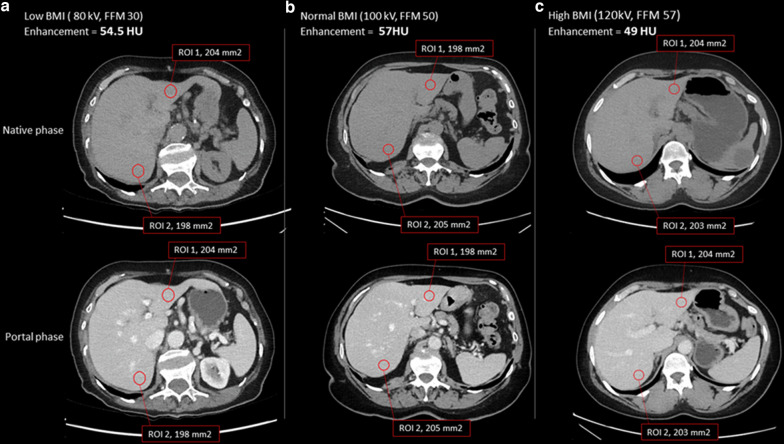
Uniform liver enhancement across different body composition and imaging parameters. Three patients with low (**a**), medium (**b**), and high (**c**) fat-free mass imaged at 80, 100, and 120 kVp, and 350/350/370 mgI/mL contrast concentration, respectively. The region of interest (ROI) analysis reveals similar liver enhancement, suggesting that the algorithm effectively adjusts for variations in body composition, imaging parameters, and contrast concentration.

**Fig. 4:**
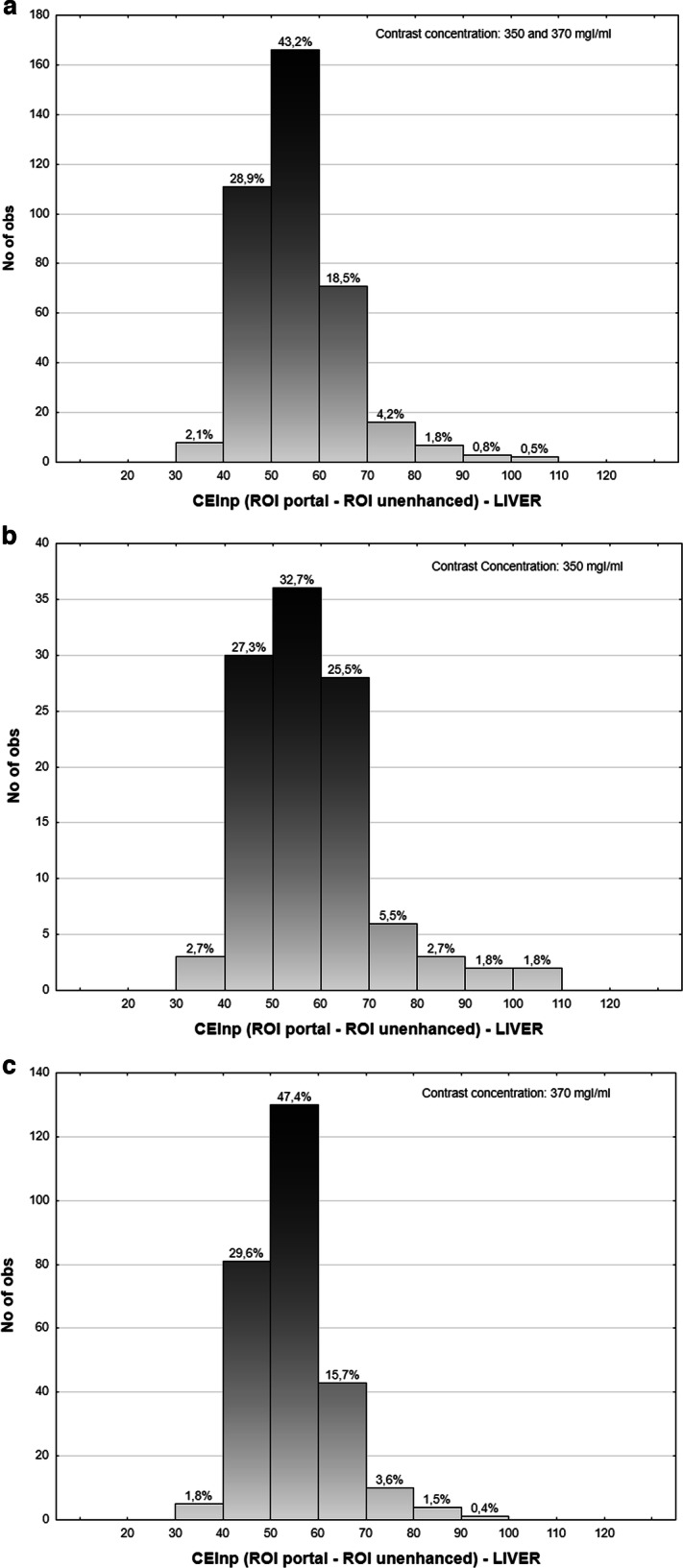
Enhancement histograms for Iopamidol and Iohexol contrast media. Histograms of the CEI_NP_ distributions for both contrast media (**a**). Histograms of the CEI_NP_ distributions for Iopamidol (350 mgI/mL) (**b**). Histograms of the CEI_NP_ distributions for the Iohexol (370 mgI/mL) (**c**). The acceptable (dotted line) and desirable (plain line) range for image quality are shown

**Fig. 5:**
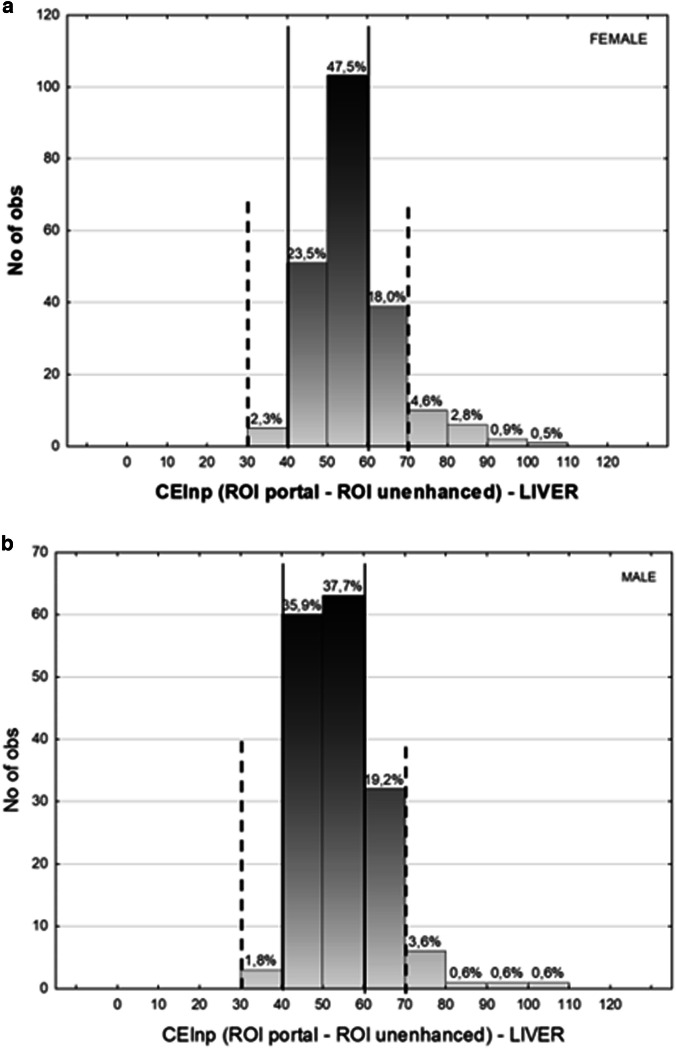
Histograms of the CEI_NP_ distributions for both contrast media. **a** Histograms of the CEI_NP_ distributions for Iopamidol (350 mgI/mL). **b** Histograms of the CEI_NP_ distributions for the Iohexol (370 mgI/mL). The acceptable (dotted line) and desirable (plain line) range for image quality are shown

Figure 3 illustrates examinations obtained from three patients with diverse body compositions (low, mean and high FFM), at different kVp and contrast agent concentrations: liver enhancement remains consistent despite these differences. Figure 4a shows that overall (both contrast agent concentrations), 92.7% of the examinations had an image quality within the acceptable range (30–70 HU), while 72.1% were within the desirable range (40–60 HU). When stratifying per contrast concentration (Fig. 4b, c), we observe that for the 350 mgI/mL concentration, the values drop to 88.2% and 60% for the acceptable and desirable image quality. For the 370 mgI/mL concentration instead, these values grow to 94.5% and 77%, respectively. This difference is however not significant (*p* = 0.123). When stratifying per sex, liver enhancement is acceptable/desirable for 91.3%/71.0% of females compared to 94.6%/73.6% of males, respectively (*p* < 0.001) (Fig. 5a, b).

## Discussion

Contrast agent injection protocols are critical for image quality, especially for the visibility and conspicuity of liver lesions in CT imaging. Harmonizing image quality implies personalizing contrast injection protocols, enabling the reproducibility of liver enhancement for diagnostic and follow-up examinations and, consequently, adequate patient management decisions. This study aimed to validate the generalizability of a novel algorithm for personalized contrast injection in adult liver CT in clinical settings, targeting a reproducible liver enhancement centered on 50 HU.

The algorithm, developed at the institution, aims to address the limitations of existing protocols that often need more generalizability. Traditional approaches are often tailored to local settings, using a specific scanner type, kVp, contrast concentration, injection rate, and total body weight-based contrast volume calculation, without targeting a reproducible liver enhancement on diagnostic or follow-up examinations across diverse patients. The algorithm’s innovation lies in its adaptability to different image quality levels, clinical settings, such as patient habitus, sex, scanner type, kVp, and contrast concentrations [15].

Indeed, patients have different shapes and sizes, which affect both imaging parameters as well as liver enhancement. The algorithm can accommodate variations in patient habitus by using FFM as a parameter, ensuring that the dosage and administration of contrast agents are appropriate for individuals regardless of their body type.

Biological differences between sexes can also influence how contrast agents are biodistributed within the body. The algorithm takes into account these differences, tailoring the contrast injection protocols to account for sex-specific variations in contrast enhancement.

Different CT scanner models may have varying automatic exposure systems, using therefore different kVp settings based on patient characteristics. The algorithm can adapt to different kVp settings, ensuring that contrast enhancement remains consistent and optimal across various scanning parameters. Contrast agents come in different concentrations and the appropriate dosage can vary depending on the concentration used. The algorithm considers the specific concentration of contrast agent being administered, calculating the dosage accordingly to achieve the desired level of enhancement. By incorporating adaptability to these diverse clinical settings, the algorithm ensures consistent and optimal contrast enhancement in CT imaging, thereby improving the accuracy and reliability of diagnostic results across a wide range of patient populations and imaging scenarios.

In this study, we evaluated the algorithm’s generalizability in a real-world clinical setting encompassing 384 patients over two years. The results indicate promising outcomes, with 92.7% of examinations falling within the acceptable image quality range (30–70 HU) and 72.1% within the desirable range (40–60 HU). No cases resulted to be underenhanced (enhancement below 30 HU) [13]. The algorithm’s effectiveness is further demonstrated by its ability to accommodate variations in contrast concentration, kVp settings, and patient sex.

This study emphasizes the importance of considering patient-specific factors. In fact, the algorithm incorporates FFM as a parameter to personalize the contrast injection protocol and predict the needed contrast agent dose for optimal liver enhancement, considering individual variations in body composition [15] As an example, Fig. 4 shows that for three patients with low, mean, and high FFM, a comparable liver enhancement, close to the target image quality (50 HU) was obtained. Our results also revealed that female patients received a significantly lower amount of iodine dose than their male counterparts, a disparity attributable to variations in body composition (females typically have a lower FFM than males). The algorithm’s adaptability ensured that liver enhancement remained within acceptable and desirable image quality ranges for both sexes.

The algorithm’s adaptability across different CT scanner generations and reconstruction technologies, including those equipped with advanced reconstruction deep-learning-based algorithms, showcases its potential for widespread implementation. Our standardized protocols across multiple centers and vendors underscore the algorithm’s versatility and applicability. As detailed in our previous study [22], our institution underwent a harmonization phase for all protocols, wherein clinical indication-based protocols were established, and acquisition and reconstruction parameters were standardized across all CT scanners. Subsequently, a dose optimization process was implemented.

Additionally, the study provides valuable insights into the impact of contrast concentration and vendor injection volume limitations. For Iohexol, the lower injection limit of 30 g initially barred the algorithm’s potential to suggest low-contrast doses, impairing the reproducibility of liver enhancement centered at 50 HU, as most livers were overenhanced. This limitation could be overcome by adapting the lower limit of contrast dose with the approval of the ethical committee. Moreover, accounting for concentration might not be enough to harmonize enhancement (see Fig. 4). Other factors such as formulation, viscosity, temperature, and specific properties of the contrast agents from each brand can influence their performance. It would be relevant to consider the pharmacokinetics and pharmacodynamics of each contrast agent.

The impact of tube potential and reconstruction methods on low-contrast liver lesion detectability [23] was integrated into the algorithm to ensure an optimized contrast dose, reducing the risk of false-negative examinations in identifying liver lesions.

The algorithm is configured to allow users to select their desired image quality (enhancement). For this study, we opted for a 50-HU enhancement level, considering it as the optimal diagnostic enhancement for liver lesion detection based on current technology available at our institution. However, it is conceivable that advancements in technology, like dual-energy CT, photon-counting detector CT, and artificial intelligence-based software, may necessitate lower liver enhancement levels in the future, and our algorithm is designed to adapt accordingly.

While we focused on validating the algorithm for personalized contrast injection in CT liver imaging, we acknowledge the importance of considering broader implications, like for example the need to counteract the shortage of iodinated contrast agents, as recently reported [24]. This situation led to the use of a reduced amount of contrast agent on a large scale, in order to stay operational. Our algorithm could also serve for this purpose, allowing, through protocol optimization, to reduce contrast dose [15].

Limitations and challenges still exist, such as adapting to patients with extreme liver conditions such as fatty liver, fibrotic changes, or hemochromatosis, and will need additional investigation. FFM measurements are also time-consuming for radiographers and currently represent a limitation in daily practice. Given the significant impact of FFM on contrast dose calculation, we have developed a theoretical model for FFM estimation, which is under validation [15].

Regarding the external validity of our findings and the generalizability to other populations and clinical settings, we believe that our algorithm can be readily adapted to different scanner types and clinical environments. To achieve this, one approach involves estimating iodine concentration scaling factors for all kVp settings using anthropomorphic phantoms, as previously demonstrated [15]. Selection bias may arise from the exclusion of certain groups such as patients with fatty liver or cirrhosis. However, this was necessary as their liver enhancement behavior substantially deviates from that of the target population, namely healthy patients. To address this exclusion, all cases excluded due to specific pathologies were grouped during data collection, with plans underway to explore these populations in a separate article. Regarding sampling bias, all patients were consecutively collected during routine clinical workflow, minimizing the risk of bias in participant selection. Furthermore, while confounding variables like contrast temperature or patient comorbidities could potentially influence contrast enhancement, any impact on the outcome may not be substantial. In fact, our aim was to achieve an enhancement centered around the point value of 50 HU, which strikes a balance between providing adequate diagnostic imaging quality without administering excessive or insufficient contrast to the patient.

In conclusion, this study presents a step forward in personalized contrast injection for liver CT, *i.e.,* a novel algorithm for personalized contrast injection in adult abdominal CT by achieving consistent liver enhancement centered on 50 HU. Its generalizability is confirmed across varying contrast concentrations, kVp settings, and patient sex, highlighting its robustness in real-world clinical applications. This algorithm offers a standardized approach that enhances diagnostic accuracy, facilitates longitudinal studies, and minimizes contrast agent usage, thereby reducing costs, environmental impact, and patient risk. Furthermore, our findings highlight the algorithm’s potential to address contrast agent shortage challenges and optimize contrast dose in response to emerging technologies.

## Data Availability

The datasets used and/or analyzed during the current study are available from the corresponding author upon reasonable request.

## Abbreviations

BMI: Body mass index
CEI: Contrast-enhancement index
CT: Computed tomography
FFM: Fat-free mass
ROI: Region of interest
